# CRISPR/Cas9-engineered *Gad1* elimination in rats leads to complex behavioral changes: implications for schizophrenia

**DOI:** 10.1038/s41398-020-01108-6

**Published:** 2020-12-08

**Authors:** Kazuyuki Fujihara, Kazuo Yamada, Yukio Ichitani, Toshikazu Kakizaki, Weiru Jiang, Shigeo Miyata, Takashi Suto, Daiki Kato, Shigeru Saito, Masahiko Watanabe, Yuki Kajita, Tomokazu Ohshiro, Hajime Mushiake, Yoshiki Miyasaka, Tomoji Mashimo, Hiroki Yasuda, Yuchio Yanagawa

**Affiliations:** 1 Department of Genetic and Behavioral Neuroscience, Gunma University Graduate School of Medicine, Maebashi City, Gunma 371-8511 Japan; 2 Department of Psychiatry and Neuroscience, Gunma University Graduate School of Medicine, Maebashi City, Gunma 371-8511 Japan; 3 Institute of Psychology and Behavioral Neuroscience, University of Tsukuba, Tsukuba City, Ibaraki 305-8577 Japan; 4 Department of Anesthesiology, Gunma University Graduate School of Medicine, Maebashi City, Gunma 371-8511 Japan; 5 Department of Anatomy, Faculty of Medicine, Hokkaido University, Sapporo City, Hokkaido 060-8638 Japan; 6 Department of Physiology, Graduate School of Medicine, Tohoku University, Sendai City, Miyagi 980-8575 Japan; 7 Institute of Experimental Animal Sciences, Graduate School of Medicine, Osaka University, Suita City, Osaka 565-0871 Japan; 8 Laboratory Animal Research Center, Institute of Medical Science, The University of Tokyo, Minato-ku, Tokyo, 108-8639 Japan; 9 Division of Physiology, Faculty of Medicine, Saga University, Saga City, Saga 849-8501 Japan

**Keywords:** Schizophrenia, Molecular neuroscience

## Abstract

GABAergic dysfunctions have been implicated in the pathogenesis of schizophrenia, especially the associated cognitive impairments. The GABA synthetic enzyme glutamate decarboxylase 67-kDa isoform (GAD67) encoded by the *GAD1* gene is downregulated in the brains of patients with schizophrenia. Furthermore, a patient with schizophrenia harboring a homozygous mutation of *GAD1* has recently been discovered. However, it remains unclear whether loss of function of *GAD1* leads to the symptoms observed in schizophrenia, including cognitive impairment. One of the obstacles faced in experimental studies to address this issue is the perinatal lethality of *Gad1* knockout (KO) mice, which precluded characterization at the adult stage. In the present study, we successfully generated *Gad1* KO rats using CRISPR/Cas9 genome editing technology. Surprisingly, 33% of *Gad1* KO rats survived to adulthood and could be subjected to further characterization. The GABA concentration in the *Gad1* KO cerebrum was reduced to ~52% of the level in wild-type rats. *Gad1* KO rats exhibited impairments in both spatial reference and working memory without affecting adult neurogenesis in the hippocampus. In addition, *Gad1* KO rats showed a wide range of behavioral alterations, such as enhanced sensitivity to an NMDA receptor antagonist, hypoactivity in a novel environment, and decreased preference for social novelty. Taken together, the results suggest that *Gad1* KO rats could provide a novel model covering not only cognitive deficits but also other aspects of the disorder. Furthermore, the present study teaches an important lesson: differences between species should be considered when developing animal models of human diseases.

## Introduction

γ-Aminobutyric acid (GABA) is a major inhibitory neurotransmitter in the mammalian central nervous system. GABA is synthesized from glutamate by two glutamate decarboxylases (GADs), 67 and 65-kDa isoforms (GAD67 and GAD65) encoded by *Gad1* and *Gad2* genes, respectively^1–3^. While GAD67 is constitutively active and accounts for basal GABA synthesis in the soma of neurons, GAD65 is transiently activated and responsible for on-demand production in the axonal terminal^4,5^. The difference in phenotypes between knockout (KO) mice of the two GADs also has indicated distinct physiological roles: the *Gad1* KO mice displayed cleft palate accompanied by perinatal lethality^6^, while the *Gad2* KO mice developed epileptic seizures in adulthood^7^.

Deficits in GABA production could also have a large impact on brain function in humans. In the cerebral cortex of subjects with schizophrenia, GAD67 mRNA^8–11^ and proteins^12^ are decreased. Conversely, GAD65 expression is unaffected^10^ or slightly reduced^13^. Interestingly, a study reported that GAD65 expression levels were reduced in schizoaffective disorder, but not in schizophrenia^14^. GAD67 downregulation is likely brain-wide because it was observed in the prefrontal cortex, parietal cortex, visual cortex^15^, and hippocampus^16^. In schizophrenia, cognitive impairments such as working memory deficits are key symptoms affecting the functional outcome of patients^17^. Because the GABAergic system plays an important role in performing working memory tasks by synchronizing neuronal activity by generating gamma oscillation^18^, GAD67 reduction is hypothesized to be a cause of cognitive impairment in schizophrenia. Accordingly, GABA concentrations in the dorsolateral prefrontal cortex have been correlated with working memory performance in healthy humans^19^.

Genetic evidence has also supported the link between the *GAD1* gene and schizophrenia. Some single nucleotide polymorphisms (SNPs) surrounding the *GAD1* locus have been associated with childhood-onset schizophrenia in a North American cohort^20^ and in ordinary schizophrenia in a Chinese cohort^21^. Among these SNPs, one was significantly associated with lower levels of GAD67 expression in postmortem analysis^22^. Some SNP variations in *GAD1* were also associated with the poorer performance of attention and working memory^23^. Furthermore, homozygous missense mutations in the *GAD1* gene were identified in a family of schizophrenia patients by whole-exome sequencing in Italy^24,25^. This mutation prevents the homodimerization of GAD67 proteins, which is necessary for their sufficient activity^25^. Based on these genetic findings combined with accumulated evidence from postmortem brain studies, we can assume that loss-of-function mutations of *Gad1* have a causal effect on the symptoms of schizophrenia, particularly on cognitive impairment. However, because the above studies are observational studies, it is unclear whether the *Gad1* mutation is the cause of the disorder.

The development of a *Gad1* knockout (KO) animal and characterization of its behavior is a reasonable strategy to test this hypothesis. However, since global *Gad1* KO is lethal in mice on the first postnatal day^6,26^, they cannot undergo behavioral testing. Researchers have also developed several conditional KO mice or knockdown (KD) mice in which GAD67 was disrupted in a restricted subpopulation of GABAergic neurons to avoid lethality^27–29^. However, these mice never exhibited any working memory impairment despite showing a few schizophrenia-related phenotypes. Do these results ultimately refute the “GAD67 hypothesis” of cognitive impairments in schizophrenia?

Animal species used for KO experiments in biomedical research have been largely restricted to mice due to technical limitations. Recently, genome editing techniques such as the clustered regularly interspaced short palindrome repeat (CRISPR)/Cas9 system^30^ have allowed researchers to develop KO or transgenic animals in various species other than mice, such as rats^31,32^, ferrets^33^, and marmosets^34^. In this situation, careful examination is necessary when selecting a species suitable for experiments^35^. Although both mice and rats belong to the order Rodentia, there are significant differences that can affect behavioral testing. First, most behavioral tests were originally developed in rats, and some of these tests are more suitable for rats than mice. For instance, in the Morris water maze test used for the assessment of spatial memory, mice have greater difficulty in learning the location of the platform compared to rats because mice tend to avoid swimming as a habit^35^. Second, rats are suggested to have a much abundant repertoire of behavioral tests for investigating cognitive functions, which is advantageous for research on psychiatric disorders^31^. This can make a difference in detecting possible cognitive impairments of *Gad1* knockout animals. In addition, with respect to GAD67 and GAD65 expression levels, a species difference has already been reported. We have previously shown that the expression levels of GAD67 and GAD65 are similar in the adult mouse brain^28^. Conversely, although the biological significance is unclear, the expression level of GAD67 is lower than that of GAD65 in rat and human brains^36,37^. Thus, the GAD67/GAD65 expression ratio in human brains is closer to that in rat brains than in mouse brains, suggesting that the rat is an invaluable experimental animal for studying the roles of GAD67 in brain function and dysfunction, in particular, the pathophysiology of schizophrenia.

Based on the above, we planned to re-examine the phenotypes caused by global and conditional *Gad1* KO in rats rather than mice. Surprisingly, we found that some global *Gad1* KO rats can survive to adulthood. Therefore, herein, we focused on the behavioral characterization of global *Gad1* KO rats to provide evidence supporting the cause-effect relationship between loss of function of GAD67 and schizophrenia-related phenotypes, including cognitive impairment. Our data will provide insight into not only the pathophysiology of the patient with the ultrarare mutation of *GAD1* but also that of other ordinary patients.

## Materials and methods

All experiments were approved by the Animal Care and Experimentation Committees of Gunma University, the Animal Research Committee of Osaka University, and the Institutional Laboratory Animal Care and Use Committee of Tohoku University. Every effort was made to minimize the number of animals used and their suffering.

### Animals

**Fig. 1:**
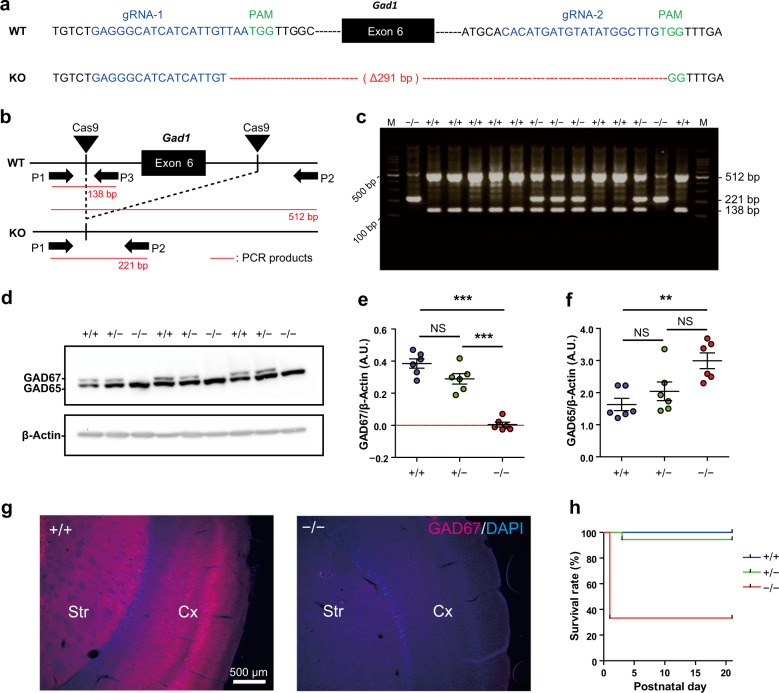
Generation of *Gad1* knockout rats by the CRISPR/Cas9 system. **a** Targeted site of *Gad1* knockout rats. WT and KO represent wild-type and knockout alleles, respectively. The two sequences highlighted in blue denote the sites recognized by the complex of Cas9 and guide RNAs (gRNA-1 and gRNA-2). The protospacer adjacent motif (PAM) sequences next to the two sites are highlighted in green. The KO allele has a 291-bp deletion, including exon-6 of the *Gad1* gene. **b** Schematic representation of CRISPR/Cas9-mediated deletion of exon-6 and the approximate locations of primers for genotyping PCR (P1, P2, and P3). **c** A representative result of genotyping PCR. M: DNA molecular weight marker. **d** Protein levels of GAD67 in the adult brain taken from each genotype (*n* = 6 in each genotype). **e** Western blot analysis using an antibody that binds both GAD65 and GAD67 revealed the loss of GAD67 protein in *Gad1*^−/−^ rats (*F*(2, 15) = 55.372, *p* < 0.001). **f** GAD65 was increased in *Gad1*^−/−^ rats (*F*(2, 15) = 8.0857, *p* < 0.01). β-Actin was used as an internal control in **d**–**f**. **g** The loss of GAD67 immunoreactivity in the *Gad1*^−/−^ rat was also observed in the immunohistochemical analysis. Cx cerebral cortex, Sr striatum. The nuclei were stained using DAPI. Scale bar = 500 μm. **h** *Gad1*^−/−^ rats showed a significantly lower survival rate than *Gad1*^+/+^ and *Gad1*^+/^^−^ rats (log-rank test, *p* < 0.001). The data were analyzed using one-way ANOVA and *post hoc* test adjusted by Holm’s method for multiple comparisons (**e**, **f**) and log-rank test (**h**). The results are presented as the average ± SEM. *p < 0.05, **p < 0.01, ***p < 0.001.

A rat line with exon-6 deletion of *Gad1* (Gene ID: 24379) was generated on a Long-Evans background (Japan SLC, Inc., Hamamatsu, Shizuoka, Japan) using previously described methods^38^. It is noteworthy that rat *Gad1* exon-6 corresponds to mouse *Gad1* exon-5^39,40^. Briefly, two CRISPR guide RNAs (gRNAs) were designed to target exon-6 of *Gad1* (Fig. 1a). To obtain both *Gad1* KO and *Gad1*-flox lines, a long single-strand DNA (lssDNA) composed of exon-6 flanked by two loxP sequences was electroporated with the two gRNAs and Cas9 mRNA into pronuclear stage embryos. The *Gad1*-flox line will be described elsewhere; herein, we focused on the *Gad1* KO line. Embryos developing into the two-cell stage after the introduction of RNAs and lssDNA were transplanted into oviducts of foster mothers. We successfully acquired an F0 rat with a 291 bp deletion, which includes exon-6 (Fig. 1a). The F0 rat was crossed with wild-type (WT) Long-Evans rat to obtain F1 heterozygous KO rats. For further experiments, we crossed heterozygous male and female rats to obtain WT (*Gad1*^+/+^), heterozygous (*Gad1*^+/^^−^), and homozygous (*Gad1*^−/−^) animals. The rats were housed in a room maintained at 22 ± 3 °C with a 12-h light-dark cycle (lights on at 6:00, lights off at 18:00). Food (CLEA Rodent Diet CE-2, Clea Japan, Meguro, Tokyo, Japan) and water were provided ad libitum.

### Genotyping PCR

The genotype of each obtained pup was determined using genomic DNA extracted from the tail tissue at P0 (to determine the survival curve) or P21–P28 (for other experiments). PCR genotyping was performed using the following primers: 5′-ACTGGGCCATTGTTCCAGCTCCA-3′ (primer 1), 5′-GCTCTCTCACGAGTATGCCCTTGCT-3′ (primer 2), and 5′-CGAGCTGGAGAAGGGGGAAGAAGAT-3′ (primer 3). Two DNA fragments of 512 bp (primer 1 and primer 2) and 138 bp (primer 1 and primer 3) were amplified from the WT allele, and a fragment of 221 bp (primer 1 and primer 2) was amplified from the KO allele (Fig. 1b, c).

### Quantification of GAD proteins and GABA in rat brain tissue

Elimination of GAD67 in *Gad1* KO rats was confirmed in cerebral cortex tissue samples taken from adult (three months old) or juvenile rats (P20–P24). GAD67 or GAD65 protein was quantified by western blot analysis as described previously^28^. Concentrations of GABA and glutamate were measured by high-pressure liquid chromatography (HPLC)^41^ in the cerebral cortex and whole cerebellum samples of rats at P20–P24 (Supplementary Information).

### Immunohistochemistry

Perfusion with 4% paraformaldehyde and immunohistochemistry were performed as described in the Supplementary materials using mouse anti-GAD67 (1:1000; Merck Millipore, Burlington, MA, USA) and anti-doublecortin (DCX) antibodies (1:1000; Merck Millipore). See Supplementary Information for details.

### Behavioral analysis

Before the behavioral tests, rats were habituated to the experimenters by a 3-day handling period. All rats were male and older than 10 weeks at the start of the behavioral analyses. We performed the Morris water maze, eight-arm radial maze, open field, novel object recognition, social interaction, Y-maze, elevated plus maze, acoustic startle response, prepulse inhibition (PPI), and forced swim tests. The eight-arm radial maze test was performed as described previously with minor modifications^42^. For the Morris water maze test, we modified the method of Nunez^43^. All other behavioral tests were carried out using a modified version of Fujihara et al.^28^ for rats. Except in the Morris water maze and radial maze tests, the genotypes of each rat were blinded to the experimenter. See the Supplementary Information for detailed procedures.

### Statistical analysis

The sample sizes of each experiment were determined based on our previous study^28^. No randomization was performed. For comparison between genotypes, we employed Welch’s *t*-test, Wilcoxon rank-sum test, one-way or two-way ANOVA with post hoc Holm’s method. Although the variances of each group were similar in most of the experiments, we applied the Welch’s *t*-test rather than Student’s *t*-test because the former is more robust to unequal variances. If the Shapiro–Wilk normality test showed a lack of normality of the data, we applied the Wilcoxon rank-sum test for group comparison. To analyze the correlations between these parameters, we calculated Spearman’s rank correlation coefficient (*rho*). For survival analysis, we used the Kaplan–Meier method with a log-rank test. We also conducted an analysis of covariance (ANCOVA) to compare the number of DCX-positive cells between the groups to adjust for age effects. *p*-values < 0.05 were considered statistically significant.

## Results

### Verification of the *Gad1* knockout

We successfully obtained a *Gad1* KO rat line, as shown in Fig. 1a–c and Supplementary Fig. 1a, b. To verify that *Gad1*^−/−^ rats expressed no GAD67 protein, we carried out Western blot analysis for the brain extract using two different antibodies (Fig. 1d and Supplementary Fig. 1b). GAD67 protein was undetectable in the *Gad1*^−/−^ rats, whereas the amount of GAD65 protein in *Gad1*^−/−^ rats was significantly upregulated to 183.5% of that in *Gad1*^+/+^ rats (Fig. 1f). A slight reduction in GAD67 protein in *Gad1*^+/−^ rats was observed but did not reach statistical significance. The elimination of GAD67 was further confirmed by immunohistochemistry in the cerebral cortex and striatum. No immunoreactivity was detected in *Gad1*^−/−^ rats (Fig. 1g).

### Lower survival rate and growth delay of *Gad1*^−/−^ rats

Each genotype of the rats was obtained at a Mendelian frequency (Supplementary Fig. 1a). The survival rate of *Gad1*^−/−^ rats was significantly lower than that of *Gad1*^+/+^ littermates, but 33% of *Gad1*^−/−^ rats grew to adulthood (Fig. 1h). The survival rate of *Gad1*^+/−^ rats was not significantly different from that of *Gad1*^+/+^ rats. This result was in contrast to cases of *Gad1*^−/−^ mice with 100% lethality at P0^6^. Furthermore, no apparent malformations, such as the cleft palate or omphalocele, were observed in *Gad1*^−/−^ mice^6,26^. *Gad1*^−/−^ rats did not show any hindlimb clasping, suggesting that they had no ataxic-like motor dysfunction (Supplementary Fig. 1c). Conversely, the body size and body weight of *Gad1*^−/−^ rats were smaller than those of *Gad1*^+/+^ rats after birth to approximately two months of age (P28: *Gad1*^+/+^, 92.5 ± 2.96 g; *Gad1*^−/−^, 59.65 ± 10.23 g; Supplementary Fig. 2), although the growth curve of homozygous *Gad1*^−/−^ rats caught up to adult rats (Supplementary Fig. 2c, d). Furthermore, the brains of *Gad1*^−/−^ rats had no obvious abnormality in gross morphology at the adult stage compared with *Gad1*^+/+^ rats (data not shown).

### Decrease in GABA concentration in *Gad1*^−/−^ rats

**Fig. 2:**
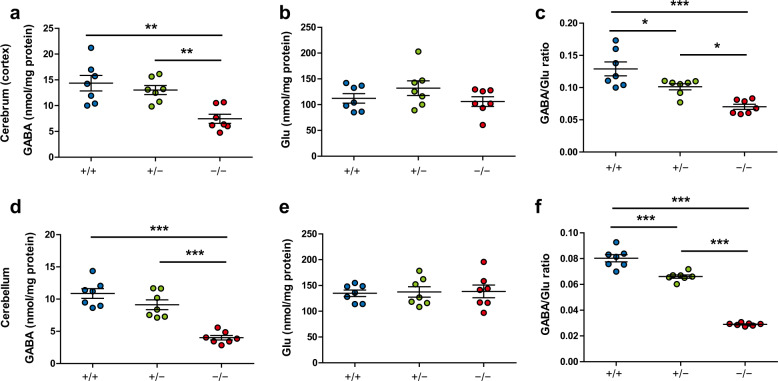
GABA and glutamate concentrations in the brains of *Gad1* knockout rats. Brain samples were collected from P20 to P24. **a** In the cerebral cortex, the GABA concentration in *Gad1*^−/−^ rats was reduced to 51.88% of that of *Gad1*^+/+^ (*F*(2, 18) = 16.748, *p* < 0.001). **b** There were no differences in the glutamate (Glu) concentrations among the three genotypes in the cerebral cortex (*F*(2, 18) = 1.4734, *p* = 0.2555). **c** The GABA/Glu ratio was reduced in both *Gad1*^+/−^ and *Gad1*^−/−^ rats in the cerebral cortex (*F*(2, 18) = 10.578, *p* < 0.001). **d** GABA concentrations in the cerebellum (*F*(2, 18) = 29.646, *p* < 0.001). **e** Glu concentrations in the cerebellum (*F*(2, 18) = 0.0372, *p* = 0.9636). **f** GABA/Glu ratios in the cerebellum (*F*(2, 18) = 218.79, *p* < 0.001). *n* = 7 in each genotype. The results are presented as the average ± SEM. The data were analyzed using one-way ANOVA and post hoc test adjusted by Holm’s method for multiple comparisons. *p < 0.05, **p < 0.01, ***p < 0.001.

The functional impact of GAD67 deletion in *Gad1*^−/−^ rats was measured by the concentration of GABA in brain tissue (Fig. 2). GABA concentrations in both the cerebral cortex and cerebellum were significantly reduced in homozygous *Gad1*^−/−^ rats (Fig. 2a, d), while *Gad1*^+/^^−^ rats showed no significant reductions. Concentrations of glutamate (Glu), the precursor of GABA, showed no differences among the three genotypes in either region (Fig. 2b, e). The GABA/Glu ratio differed among all genotypes in both the cerebral cortex and cerebellum (Fig. 2c, f).

### Reduced locomotion and rearing in *Gad1*^−/−^ rats

**Fig. 3:**
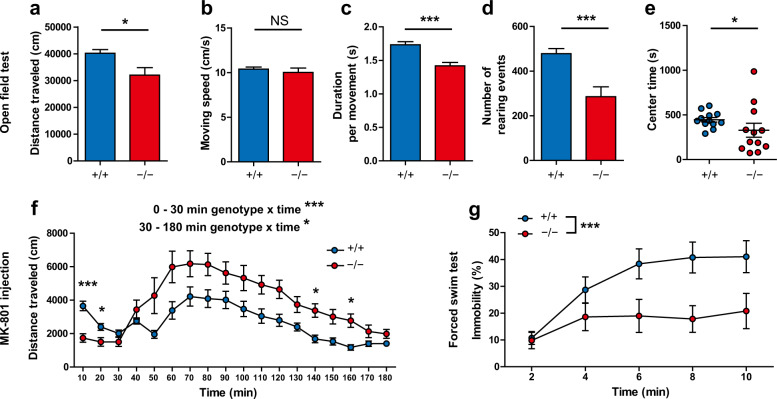
Task-dependent alterations of activities in *Gad1*^−/−^ rats. **a**–**e** Open field test (*n* = 12 for each genotype). *Gad1*^−/−^ rats exhibited decreased distance traveled compared with *Gad1*^+/+^ (*t*(16.051) = –2.6272, *p* < 0.05) (**a**), but their moving speed was unaltered (*t*(15.717) = −0.6772, *p* = 0.5081) (**b**). Duration per movement (*t*(21.82) = –4.541, *p* < 0.001) (**c**) and number of rearing events (*t*(16.456) = −3.8561, *p* < 0.01) (**d**) were also decreased in *Gad1*^−/−^ rats, suggesting hypoactivity in the open field. *Gad1*^−/−^ rats stayed less time in the center region (*W* = 37, *p* < 0.05) (**e**). **f** Response to NMDA receptor antagonist (*Gad1*^+/+^, *n* = 12; *Gad1*^−/−^, *n* = 11). *Gad1*^−/−^ rats showed hypoactivity at baseline (0–30 min; genotype × time, *F*(2, 40) = 9.3185, *p* < 0.001) but showed enhanced hyperactivity after MK-801 injection (0.2 mg/kg, intraperitoneal injection) (30–180 min; genotype × time, *F*(14, 280) = 1.9404, *p* < 0.05; simple main effect of genotype, adjusted by Holm’s method for multiple comparisons; 130–140 min, *p* < 0.05; 150–160 min, *p* < 0.05). **g** Forced swim test (*Gad1*^+/+^, *n* = 12; *Gad1*^−/−^, *n* = 11). *Gad1*^−/−^ rats showed a significant decrease in immobility (genotype main effect, *F*(1, 20) = 6.4689, *p* < 0.05). The results are presented as the average ± SEM. The data were analyzed using the Wilcoxon rank-sum test (**e**), two-way repeated measures ANOVA (**f**–**h**), and Welch’s *t*-test (others). *p < 0.05, **p < 0.01, ***p < 0.001; NS not significant.

Behavioral consequences of *Gad1* deletion were determined by behavioral testing in *Gad1*^−/−^ rats and *Gad1*^+/+^ littermates. In the open field test for seven days, *Gad1*^−/−^ rats showed reduced distance traveled compared with *Gad1*^+/+^ rats (Fig. 3a), although their moving speed was comparable to *Gad1*^+/+^ rats (Fig. 3b). The duration per movement and the number of rearing events were also reduced in *Gad1*^−/−^ rats (Fig. 3c, d). Figure 3 reports the 7-day summations for each parameter. The time course of each parameter is shown in Supplementary Fig. 3. As a whole, *Gad1*^−/−^ rats were characterized by hypoactive behavior.

The exploration time in the center region (center time) and number of fecal boli in the open field were measured. Because the data of the exploration time in the center region (center time) in *Gad1*^−/−^ rats showed neither normality (*W* = 0.845, *p* < 0.05) nor equality of variance to *Gad1*^+/+^ rats (*F*(11, 11) = 9.1871, *p* < 0.001), a nonparametric test was used for comparisons. There was a significant decrease in the center time in *Gad1*^−/−^ rats (Fig. 3e; Supplementary Fig. 4a). The total number of fecal boli in the open field session also increased in *Gad1*^−/−^ rats (Supplementary Fig. 4b) and negatively correlated with the center time and distance traveled only in *Gad1*^−/−^ rats (Supplementary Fig. 4c, d).

### *Gad1*^−/−^ rats showed no alterations in the elevated plus-maze test

We assessed the behavior of *Gad1*^−/−^ rats on an elevated plus-maze. However, we did not detect any alterations, such as reduced exploration of the open arms of the maze (Supplementary Fig. 5).

### Enhanced sensitivity to NMDA receptor antagonists in *Gad1*^−/−^ rats

Treatment with NMDA receptors can elicit schizophrenia-like symptoms in humans and schizophrenia-like behaviors in rodents^28,44^. Therefore, we analyzed the sensitivity to the NMDA antagonist MK-801 (0.2 mg/kg, intraperitoneal injection) (Fig. 3f). During the habituation phase of the open field arena before administration of MK-801, *Gad1*^−/−^ rats showed significantly lower locomotor activity. Following MK-801 injection, the moving distance in both genotypes increased from baseline. Meanwhile, the distance traveled by *Gad1*^−/−^ rats was significantly higher than that traveled by *Gad1*^+/+^ rats. Subsequent *post hoc* tests confirmed the enhanced hyperlocomotion induced by MK-801 in *Gad1*^−/−^ rats compared with *Gad1*^+/+^ rats.

### *Gad1*^−/−^ rats exhibited reduced immobility in the forced swim test

*Gad1*^−/−^ rats also showed enhanced activity in the forced swim test. In the test session, *Gad1*^−/−^ rats became significantly less immobile than *Gad1*^+/+^ rats (Fig. 3g).

### Normal acoustic startle response and sensorimotor gating in *Gad1*^−/−^ rats

Deficits in sensorimotor gating are a translatable endophenotype of schizophrenia^28^. We tested possible deficits in the startle response and sensorimotor gating using an acoustic startle response, yet there were no differences between *Gad1*^−/−^ and *Gad1*^+/+^ rats (Supplementary Fig. 6).

### Reduced social novelty preference in *Gad1*^−/−^ rats with intact object recognition

**Fig. 4:**
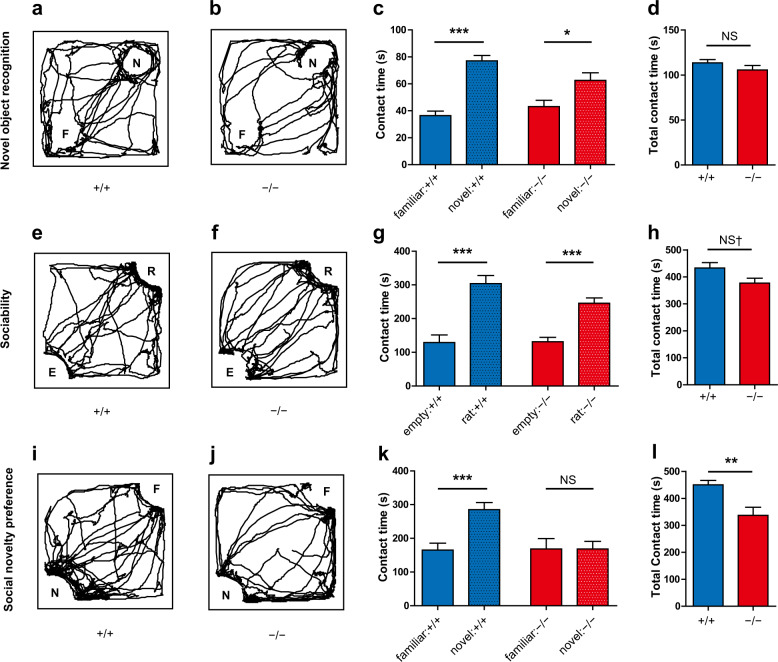
*Gad1*^−/−^ rats showed a deficit in social novelty preference with intact novel object recognition. **a**–**d** Novel object recognition task (*Gad1*^+/+^, *n* = 12; *Gad1*^−/−^, *n* = 11). Representative trajectories of *Gad1*^+/+^ (**a**) and *Gad1*^+/+^ (**b**) rats in the arena. N: novel object, F: familiar object. Both *Gad1*^+/+^ and *Gad1*^−/−^ rats showed longer contact with the novel object than with the familiar object (*Gad1*^+/+^, *t*(21.351) = –7.696, *p* < 0.001; *Gad1*^−/−^, *t*(19.277) = –2.6428, *p* < 0.05) (**c**). Total contact time (novel + familiar) was similar between the two genotypes (*t*(18.949) = 1.257, *p* = 0.2240) (**d**). **e–h** Sociability test (*Gad1*^+/+^, *n* = 12; *Gad1*^−/−^, *n* = 11). Representative trajectories of *Gad1*^+/+^ (**e**) and *Gad1*^−/−^ (**f**) rats are shown. R: rat, E: empty. *Gad1*^−/−^ rats preferred the cage enclosing another rat to the empty cage, similar to *Gad1*^+/+^ rats (*Gad1*^+/+^, *t*(21.908) = 5.2842, *p* < 0.001; *Gad1*^−/−^, *t*(19.447) = –5.5212, *p* < 0.001) (**g**). However, the total contact time (rat-occupied cage + empty cage) of *Gad1*^−/−^ rats decreased slightly (*t* (20.92) = 2.0506, *p* = 0.05304) (**h**). **i–l** Social novelty preference test (*Gad1*^+/+^, *n* = 12; *Gad1*^−/−^, *n* = 11). The trajectory of *Gad1*^+/+^ rats accumulated around the novel stranger rat (**i**). N: novel rat, F: familiar rat. This trend diminished in *Gad1*^−/−^ rats (**j**). *Gad1*^−/−^ rats spent significantly less time interacting with the novel rat than *Gad1*^+/+^ rats (*Gad1*^+/+^, *t*(21.991) = −4.1077, *p* < 0.001; *Gad1*^−/−^, *t*(18.266) = –0.0005, *p* = 0.9996) (**k**). In addition, the total contact time (novel rat + familiar rat) was significantly lower in *Gad1*^−/−^ rats (*t*(15.604) = 3.3153, *p* < 0.01) (**l**). The results are presented as the average ± SEM. Data were analyzed using Welch’s *t*-test. ^†^*p* < 0.1, *p < 0.05, **p < 0.01, ***p < 0.001; NS not significant.

We assessed the short-term recognition memory of *Gad1*^−/−^ rats using the novel object recognition task^45^. Both *Gad1*^−/−^ and *Gad1*^+/+^ rats explored the novel object longer than the familiar object (Fig. 4a–c). The total contact time to the objects (novel object + familiar object) was also comparable between the two genotypes (Fig. 4d). Thus, *Gad1*^−/−^ rats had no obvious deficit in recognition memory, at least discriminating between two objects.

We also tested rats for sociability and social novelty preference using the same apparatus as the novel object recognition test. In the sociability test for social vs. empty preferences, both genotypes of rats spent more time interacting with the rat-occupied cage than with the empty cage (Fig. 4e–g). *Gad1*^−/−^ rats showed slightly reduced total contact times (rat-occupied cage + empty cage) but at a subthreshold level (Fig. 4h). Subsequently, we examined social novelty preferences, which are relevant to social memory. While *Gad1*^+/+^ rats spent more time interacting with the novel rat than with the familiar rat (Fig. 3i, k), *Gad1*^−/−^ rats did not display such a preference (Fig. 4j–l). Furthermore, *Gad1*^−/−^ rats showed significantly reduced total contact times (novel rats + familiar rats) (Fig. 4l).

### *Gad1*^−/−^ rats exhibited spatial cognitive impairments and aberrant hyperactivity

**Fig. 5:**
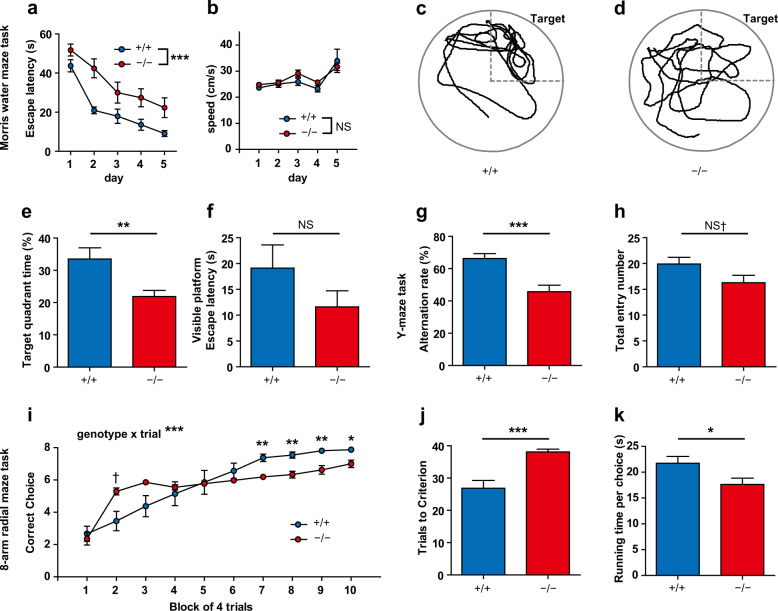
Spatial cognitive impairments in *Gad1*^−/−^ rats. *Gad1*^−/−^ rats displayed significant cognitive impairments in multiple tasks. **a**–**f** Morris water maze task (*Gad1*^+/+^, *n* = 12; *Gad1*^+/+^, *n* = 12). *Gad1*^−/−^ rats required a longer time to reach the hidden platform during the 5-day training period (main effect of genotype, *F*(1, 22) = 20.4574, *p* < 0.001) (**a**). However, the swimming speed of the *Gad1*^−/−^ rat was very similar to that of the *Gad1*^+/+^ rat (main effect of genotype, *F*(1, 22) = 0.5307, *p* = 0.4740) (**b**). Representative trajectories of *Gad1*^+/+^ (**c**) and *Gad1*^−/−^ (**d**) rats in the probe test. The target quadrant is shown in the panels. In *Gad1*^−/−^ rats, the accumulation of the trajectory to the target was decreased. The percentage of time spent within the target quadrant was significantly reduced in *Gad1*^−/−^ rats compared with *Gad1*^+/+^ rats (*t*(17.033) = −2.9599, *p* < 0.01) (**e**). There was no significant difference between genotypes in the visible platform task (*t*(19.591) = −1.3764, *p* = 0.1842) (**f**). **g**–**h** Y-maze test for working memory (*Gad1*^+/+^, *n* = 12; *Gad1*^+/+^ *n* = 11). *Gad1*^−/−^ rats showed less alternation behavior than *Gad1*^+/+^ rats (*t*(18.877) = –4.0583, *p* < 0.001) (**g**). The number of entries to arms was also slightly decreased in *Gad1*^−/−^ rats (*t*(20.453) = –1.8815, *p* = 0.07422) (**h**). **i**–**k** Eight-arm radial maze task for working memory (*Gad1*^+/+^, *n* = 12; *Gad1*^−/−^, *n* = 12). *Gad1*^−/−^ rats showed a different pattern in the learning curve from that of *Gad1*^+/+^ rats (genotype × trials, *F*(9, 198) = 9.1844, *p* < 0.001). At the end of the training, the number of correct choices was less in *Gad1*^−/−^ rats than in *Gad1*^+/+^ rats (simple main effects of genotype, adjusted by Holm’s method; 7th to 9th, *p* < 0.01; 10th, *p* < 0.05). However, in the second block, *Gad1*^−/−^ rats transiently scored higher than WT rats (2nd, *p* = 0.05227) (**i**). *Gad1*^−/−^ rats also needed more trials to reach the learning criterion of the task (*t*(13.78) = 4.3409, *p* < 0.001) (**j**). Running time per choice was decreased in *Gad1*^−/−^ rats (*t*(21.868) = 2.3065, *p* < 0.05) (**k**). The results are presented as the average ± SEM. Data were analyzed using two-way repeated measures ANOVA (**a**, **b**, **i**) and Welch’s *t*-test (**e**–**h**, **j**, **k**). ^†^*p* < 0.1, *p* < 0.05, **p < 0.01, ***p < 0.001; NS, not significant.

To assess the spatial reference memory of *Gad1*^−/−^ rats, the hidden platform task in the Morris water maze was used. During task training, the latency to escape of *Gad1*^−/−^ rats was significantly longer than that of *Gad1*^+/+^ rats (Fig. 5a). However, the swimming speed of *Gad1*^−/−^ rats was unaltered (Fig. 5b). Furthermore, in the probe test the day after the end of the training, *Gad1*^−/−^ rats stayed less in the target quadrant (Fig. 5c–e). In the visible platform test, however, there was no significant difference in the escape latency between the two genotypes (Fig. 5f).

Spatial working memory was measured using the Y-maze task (Fig. 5g, h) and the eight-arm radial maze task (Fig. 5i–k). *Gad1*^−/−^ rats showed reduced spontaneous alternation behavior in the Y-maze task (Fig. 5g). Total entry number to arms, an indicator of locomotor activity on the maze, showed a trend of reduction in *Gad1*^−/−^ rats, albeit the difference was not statistically significant (Fig. 5h).

In the acquisition curve of the eight-arm radial maze task, there was a significant genotype×trial interaction on the number of correct choices (Fig. 5i). The post hoc test revealed that the performance of *Gad1*^−/−^ rats was significantly poorer than that of WT rats during the 7th to 10th block of training. The number of trials needed to meet the learning criterion (correct choice ≥7 for five consecutive trials) was also prolonged (Fig. 5j). Conversely, *Gad1*^−/−^ rats showed slightly higher scores on the 2nd block (Fig. 5i). However, this did not indicate that *Gad1*^−/−^ rats performed well because their score was similar to the chance-level value of the radial maze task (~5.3)^46^. This was caused by increased running speed from the early stage of training in *Gad1*^−/−^ rats (see below). A similar acquisition curve was observed in a pharmacological model of schizophrenia in our previous study^42^. The running time per choice^42^, which reflects the running speed of rats during the task, was significantly shortened in *Gad1*^−/−^ rats (Fig. 5k). The faster a rat runs on the maze, the smaller the running time becomes. Therefore, the results indicate that *Gad1*^−/−^ rats moved faster than *Gad1*^+/+^ rats in the radial maze task, unlike in the open field test and the Y-maze task.

### Adult neurogenesis in the dentate gyrus was not altered in *Gad1*^−/−^ rats

The results from the behavioral tests strongly suggest that *Gad1*^−/−^ rats had spatial cognitive deficits, which can be affected by abnormalities in adult neurogenesis. GABAergic transmission plays a pivotal role in regulating adult neurogenesis^47^. Furthermore, alterations in adult neurogenesis have been reported in some animal models of schizophrenia^48^. DCX is known as one of the molecular markers of neurogenesis^48^. Therefore, we investigated possible impairments of adult neurogenesis in the hippocampus, where we counted DCX-positive cells in the dentate gyrus of *Gad1*^−/−^ and *Gad1*^+/+^ rats (Supplementary Fig. 7a, b). Both genotypes showed an age-dependent reduction in the number of DCX-positive cells. After adjustment for age, there was no difference in the number of DCX-positive cells (Supplementary Fig. 7c).

## Discussion

To test the hypothesis that loss-of-function mutation in the *GAD1* gene leads to cognitive impairments observed in schizophrenia, we generated *Gad1* KO rats using genome editing. Despite the relatively high neonatal mortality and transient growth retardation during the developmental stage, behavioral tests were feasible in adulthood, unlike *Gad1*^−/−^ mice^6^. In line with our hypothesis, we found significant impairments in both spatial reference and working memory in adult male *Gad1*^−/−^ rats. To our knowledge, this is the first direct evidence that elimination or decrease in GAD67 expression can cause distinct impairments in spatial memory in particular. Since we also identified behavioral alterations alongside cognitive impairments, *Gad1*^−/−^ rats may recapitulate a broader range of symptoms of schizophrenia.

How does loss of GAD67 expression lead to spatial cognitive impairments? The Morris water maze, eight-arm radial maze, and Y-maze tasks are known as hippocampus-dependent tasks^49–51^. Although adult neurogenesis plays distinct roles in hippocampus-dependent functions such as cognition^52^, we did not detect any reduction in adult-born neurons in the *Gad1*^−/−^ hippocampus. Thus, the poorer performance observed in these tasks must be independent of hippocampal adult neurogenesis. CA1 and hilar GABAergic neurons are required for spatial working memory and spatial reference memory, respectively^53,54^. Therefore, cognitive impairments in *Gad1*^−/−^ rats may be partially explained by loss of GAD67 and subsequent impaired GABAergic transmission. However, conditional KO or KD (cKO/KD) of *Gad1*, which mainly targets parvalbumin (PV)-positive GABAergic neurons, causes no impairment of spatial working memory in mice^27–29,55^. PV neurons are the largest population of GABAergic neurons^56,57^ and are preferentially impaired in schizophrenia^12^. In mice, acute optogenetic suppression of PV neurons in the hippocampus impairs working memory^53^, which is consistent with the present study but is inconsistent with the results of cKO/KD mice. The optogenetic technique probably can suppress GABAergic transmission more strongly than the genetic reduction of GAD67 because the release of GABA produced by GAD65 is also inhibited. Different effects on working memory between our global *Gad1* KO rats and the cKO/KD mice also may be attributed to differences in the severity of cognitive impairment. All previous studies of cKO/KD mice employed only spontaneous alternation behavior on the Y-maze for evaluation of spatial working memory^27–29,55^. However, the number of arms to be memorized is considerably fewer in the Y-maze than in the eight-arm radial maze. In addition, spontaneous alternation is suggested to be less sensitive for detecting working memory impairment than the eight-arm radial maze task in some cases^58^. Compared to global KO rats, GAD67 reduction in cKO/KD mice is considered to be mild. If the working memory impairment in the mice was also moderate, it would be difficult to detect it merely by spontaneous alternation. Furthermore, species differences in the functional role of GAD67 between mice and rats should also be considered as the cause of the discrepancy, although the significance of species differences in the GAD67/GAD65 ratio has yet to be determined. *Gad1* cKO/KD rats are needed in the future to explore species differences more directly. Since we have generated a *Gad1*-flox rat line, this will be our focus in subsequent studies.

In addition to cognitive impairments, *Gad1*^−/−^ rats showed characteristic alterations in activity. In the open field test, spontaneous locomotion and rearing were significantly reduced, while NMDA receptor antagonist-induced hyperlocomotion was enhanced compared with *Gad1*^+/+^ rats. We must carefully interpret the reduced activity in the context of schizophrenia research using animal models. One possible interpretation is that hypoactivity is a model of negative symptoms. Patients with chronic schizophrenia display increased resting time during daily activities, and the extent of their activity is negatively correlated with the severity of negative symptoms^59^. Even after the 7-day habituation period in the open field, *Gad1*^−/−^ rats still displayed a shorter duration of movement, i.e., a longer resting period. Therefore, the lower baseline activity of *Gad1*^−/−^ rats may represent a negative symptom-like phenotype. *Gad1*^−/−^ rats also displayed reduced social novelty preference, which is considered indicative of negative symptoms or impaired social recognition memory^28^. Because *Gad1*^−/−^ rats could perform normally in the novel object recognition task, this alteration is specific to the social context. Another speculation for hypoactivity is that it is a manifestation of increased anxiety in *Gad1*^−/−^ rats. We observed significant correlations among the center time, number of fecal boli, and locomotor activity in *Gad1*^−/−^ rats. The decrease in exploration time of the center region of the open field^60^ and increased number of fecal boli^61^ are considered anxiety-like behaviors in rodents. The enhanced anxiety-like phenotype is partially consistent with somatostatin neuron-specific *Gad1* KO mice, which showed a decrease in the center time during the open field test^62^. Conversely, no differences between the two genotypes were noted in the elevated plus-maze test. Therefore, caution is warranted when making conclusions about anxiety levels at present.

In contrast to hypoactivity in the open field test, hypersensitivity to NMDA receptor antagonists is relatively easy to interpret as a schizophrenia symptom. This phenotype is considered a hallmark of animal models of positive symptoms^28^ and is suggested to be mediated by the dopaminergic system^63^. Therefore, our results suggest that the decrease in GAD67 levels also influences the positive symptom-like phenotype in rats. It will be of interest to determine whether dopamine levels are altered following the administration of an NMDA receptor antagonist or amphetamine in *Gad1*^−/−^ rats. Because this phenotype is shared with PV neuron-specific *Gad1* heterozygous KO mice^28^, as we have reported previously, it is also likely to be mediated by dysfunction of this subtype of GABAergic neurons.

*Gad1*^−/−^ rats also share some phenotypes with pharmacological models of schizophrenia. Neonatal repetitive administration of NMDA receptor antagonists, such as MK-801 and ketamine, are pharmacological models of schizophrenia^42,58,64,65^. MK-801-treated rats showed significantly decreased rearing or a trend-level reduction in locomotor activity as adults, similar to *Gad1*^−/−^ rats^42,58,65^. Surprisingly, these rats displayed hyperactivity in the eight-arm radial maze task^42^ and decreased immobility in the forced swim test after stress^58^. Although the neurobiological basis of these behavioral manifestations in NMDA receptor antagonist-treated rats remains to be determined, some common mechanisms may exist between *Gad1*^−/−^ rats and NMDA receptor antagonist-treated rats. Notably, blockade of NMDA receptors during the postnatal period induces a marked reduction in PV-positive GABAergic neurons^66^ and GAD67 protein^67^. Assuming that GABAergic dysfunction itself is the common pathway of the behavioral alterations, the *Gad1*^−/−^ rat model represents a helpful tool to reveal the downstream phenomena of repetitive postnatal NMDA receptor blockade.

This study also indicated that *Gad1*^−/−^ rats failed to show deficits in PPI, an intermediate phenotype in models of schizophrenia widely assessed in animal studies^28,65^. Because GAD65 elimination causes a robust deficit of PPI in mice^68^, GABAergic transmissions have been implicated in normal sensorimotor gating. However, interestingly, GAD67 elimination showed no alterations in PPI in rats. According to postmortem brain studies, GAD65 expression is unchanged in schizophrenia patients^11^, although we observed that GAD65 expression was markedly upregulated in *Gad1*^−/−^ rat brains. The intact PPI may be attributed to this molecular discrepancy between the patients and *Gad1*^−/−^ rats.

Combined with studies on *Gad1*^−/−^ mice^7^, the present study revealed evident differences between mice and rats with loss-of-function mutations in *Gad1*. In terms of mortality, the *Gad1*^−/−^ phenotype in rats appears to be less severe than that of *Gad1*^−/−^ mice. Although the biological significance of the species difference in the GAD67/GAD65 ratio remains elusive, the reduced dependence on GAD67 in rats would enable them to survive to adulthood even if this isoform was lost. Furthermore, the cleft palate observed in *Gad1*^−/−^ mice was absent in *Gad1*^−/−^ rats, which implies that the lower GAD67/GAD65 ratio in rats prevented palate malformation as well. Meanwhile, we unexpectedly discovered a reduced body weight of *Gad1*^−/−^ rats only during development. Although some evidence suggests that GABA has important roles in controlling food intake and secretion of growth hormone^69–74^, the precise mechanisms of growth retardation in *Gad1*^−/−^ rats should be addressed to exclude potential effects on brain development and behavior. Notably, GAD67 elimination reduced GABA levels to almost half those in *Gad1*^+/+^ rat brains (Fig. 2), although the GAD67 protein amount accounted for only 23% of the total GAD proteins in the *Gad1*^+/+^ rat brain^36^, and GAD65 showed compensatory upregulation in *Gad1*^−/−^ rats (Fig. 1f). These findings indicate that GAD67 is important for maintaining baseline GABA levels in rats. Asada et al.^6^ speculated that the cause of death in *Gad1*^−/−^ mice was respiratory failure rather than cleft palate. The lethality without cleft palate observed in a subpopulation of *Gad1*^−/−^ rats also supports their hypothesis.

We also need to acknowledge some limitations of the current study. First, the *Gad1*^−/−^ rats are just a model of an ideal situation wherein GAD67 is completely eliminated. As reported in the postmortem brain studies, GAD67 is not completely lost in schizophrenia. GAD67 mRNA levels are only reduced by 15–35% when compared to the unaffected population^9,11,12^. Moreover, this reduction occurs largely in the PV-positive GABAergic neurons^12^. The effect of milder reduction and cell type-specific knockout of GAD67, as well as their interaction with environmental factors, should be analyzed in future studies. Second, although some studies have discovered the association between *GAD1* and schizophrenia, a recent genome-wide association study in a larger population did not report *GAD1* as a susceptibility gene^75^. Therefore, it should be noted that the evidence of an association between the genetic variation of *GAD1* and schizophrenia is limited. We also would like to emphasize that the *Gad1*^−/−^ rats are a model for revealing the effects of GAD67 reduction and not a model that exactly mimics the genetic variation of *GAD1* discovered in patients.

In conclusion, the loss-of-function mutation in the *Gad1* gene causes not only cognitive impairments but also several behavioral alterations possibly relevant to positive and negative symptoms of schizophrenia. *Gad1*^−/−^ rats will represent a novel tool to study pathophysiology and to develop treatments for cognitive impairment in schizophrenia. Furthermore, the results of our study warn researchers to pay closer attention to potential species differences between mice and rats when developing animal models of human disorders.

## Supplementary information

Supplementary Information
