# Supplementary material for: CRISPR/Cas9-engineered Gad1 elimination in rats leads to complex behavioral changes: implications for schizophrenia
Source: Transl Psychiatry. 2020 Dec 8;10:426. doi: 10.1038/s41398-020-01108-6 (PMC7723991; doi:10.1038/s41398-020-01108-6)
Supplement: Supplementary file 1 — Supplementary Information [file 41398_2020_1108_MOESM1_ESM.pdf]

Supplementary Information

CRISPR/Cas9-engineered *Gad1* elimination in rats leads to complex behavioral changes: Implications for schizophrenia

Kazuyuki Fujihara, MD, PhD; Kazuo Yamada, PhD; Yukio Ichitani, PhD; Toshikazu Kakizaki, PhD; Weiru Jiang, MS; Shigeo Miyata, PhD; Takashi Suto, MD, PhD; Daiki Kato, MS; Shigeru Saito, MD, PhD; Masahiko Watanabe, MD, PhD; Yuki Kajita, PhD; Tomokazu Ohshiro, PhD; Hajime Mushiake, MD, PhD; Yoshiki Miyasaka, MS; Tomoji Mashimo, PhD; Hiroki Yasuda, MD, PhD; Yuchio Yanagawa, MD, PhD

1. Supplementary Materials and Methods

1.1. Western blot analysis

We performed Western blot analysis as described previously with minor modifications[1]. Brain tissues were taken from adult (three months old) or juvenile rats (P20–P24) and then homogenized in ice-cold homogenization buffer (320 mM sucrose, 50 mM Tris-HCl [pH 7.2], 5 mM EDTA). A tablet of protease inhibitor cocktail cOmplete EDTA-free (#04693132001, Roche Diagnostics, Mannheim, Germany) was added to the buffer following the manufacturer's instructions. The homogenates were centrifuged at 1000 × g for 10 min at 4°C, and the S1 fractions were collected. The protein concentrations were measured using a TaKaRa BCA Protein Assay Kit (#T9300A, Takara Bio Inc., Shiga, Japan). The proteins (10 μg) were separated by 8% sodium dodecyl sulfate-polyacrylamide gel electrophoresis. The proteins were then transferred to a nitrocellulose membrane using a semidry transfer method. GAD67 protein was detected using mouse anti-GAD67 monoclonal antibody (1:1000, #MAB5406, Millipore, Burlington, MA, USA) or rabbit anti-GAD65/67 antibody (1 μg/mL)[1,2]. Mouse anti-β-actin antibody (1:10000, #AC-15 [ab6276], Abcam, Cambridge, United Kingdom) was used to detect β-actin as an internal control. After incubation with HRP-conjugated secondary antibody, proteins on the membrane were visualized with the Enhanced Chemiluminescence (ECL) Western blotting Analysis System (#RPN2209, GE

Healthcare Life Sciences, Buckinghamshire, UK) and imaged with the Light-Capture system (ATTO, Tokyo, Japan). The intensities of the signals of GAD67 and GAD65 were quantified using ImageJ software (https://imagej.net/) and normalized to that of β-actin.

1.2 Quantification of GABA and glutamate by HPLC

The cerebral cortex and whole cerebellum were taken from rats during P20–P24. They were then homogenized in an identical buffer for Western blot analysis. The obtained S1 fractions were diluted with a 10-fold volume of methanol and centrifuged at 15000 × g for 15 min at 0°C. After filtering with Ultrafree-MC GV 0.22 μm (#UFC30GVNB, Merck Millipore, Burlington, MA, USA), the samples were diluted again to adjust the concentration of methanol to 10%. GABA and glutamate contents in the samples were measured by an HPLC system with electrochemical detection (HTEC-500, EICOM CO., Kyoto, Japan) as described previously[3].

1.3 Immunohistochemistry for GAD67

We performed immunohistochemistry as described previously[1]. Deeply anesthetized adult rats were perfused with PBS and 4% paraformaldehyde. The brains of the rats were removed immediately after perfusion and were then immersed in 4% paraformaldehyde for 12 h. After fixation, the brains were transferred to 30% sucrose in PBS. More than 2 days later, the

brains were embedded in OCT compound (Sakura Finetek Japan, Tokyo, Japan). The embedded tissues were frozen immediately with dry ice and stored at –80°C until the experiment. Cryosections of 50 μm thickness were prepared by cryostat (Leica Biosystems, Wetzlar, Germany) and incubated with 1% bovine serum albumin for 1 h at room temperature. They were then incubated with primary antibodies in PBS for 12 h as follows: mouse anti-GAD67 (1:1000, #MAB5406, Millipore, Burlington, MA, USA). The sections were washed in PBS and incubated for 2 h with Alexa Fluor 594-conjugated secondary antibodies diluted 1:200 in PBS. The sections were also costained with DAPI before imaging. The stained sections were observed with a BZ-X810 fluorescence microscope (Keyence, Osaka, Japan) at Gunma University. In addition, we performed immunostaining for doublecortin (DCX) in the hippocampus. The procedure is described in section **1.4**.

1.4. Immunohistochemical analysis of doublecortin (DCX)

For immunohistochemical analysis of the hippocampus, the brains were sectioned at a thickness of 30 μm. The sections were stocked in 12-well plates for the floating method. The sections were pretreated with 0.1% Triton X-100 in PBS for 15 min and washed with PBS. After blocking with 3% bovine serum albumin in PBS for 1 h, the sections were incubated with primary antibodies overnight at 4°C and washed with PBS. They were then incubated with appropriate species-specific secondary antibodies for 1 h. The antibodies used in this

study were as follows: guinea pig polyclonal anti-doublecortin (DCX) antibody (1:1000; Merck Millipore, Burlington, MA, USA), Alexa Fluor 647-conjugated goat anti-guinea pig IgG, and 4',6-diamidino-2-phenylindole, dihydrochloride (DAPI) solution (1: 500, Dojindo, Kamimashiki, Japan).

Fluorescence images of cells were obtained under a microscope with a Plan-Apochromat 20x/0.80/Dry/DIC (WD = 0.55 mm) objective lens (LSM 800, Carl Zeiss, Oberkochen, Germany). Images were captured using a charge-coupled device (CCD) camera (AxioCam MRm, Carl Zeiss), transferred to a computer, and analyzed using ZEN software (Carl Zeiss). Eight sections of the dentate gyrus were analyzed per animal, and the average number was used for statistics. Cell counting was performed in a blind manner.

1.5. Behavioral analysis

1.5.1. Morris water maze test

We modified the method of Nunez for the Morris water maze test[4,5]. A white circular pool 150 cm in diameter (O'Hara, Tokyo, Japan) was filled with water. The temperature of the water was maintained at 22 ± 2° C during the test. The pool was divided into four quadrants by 90 degrees. A platform made of transparent Plexiglas (20 cm diameter) was placed in one of the quadrants. Each rat was assigned randomly to one of the quadrants and then trained to

swim to the platform from the assigned start point. A training of 1 min duration was repeated 4 times a day for 5 consecutive days. The behavior of the rats was recorded by a CCD camera. On the next day of the training, we removed the platform to carry out the probe test. In the probe test, each rat was put in the water and allowed to swim freely for 1 min. We recorded the time each rat spent inside the quadrant where the platform had been placed during the training. On the same day, we also carried out the visible platform test to verify whether their vision and motor skill were intact. A hexahedron, which was visible for the rats, was attached on the platform, and then the time to escape to the platform was recorded for each rat.

1.5.2. Radial maze test

An elevated eight-arm radial maze[6] designed by one of the authors (Y.I.) was used as described previously[7]. Briefly, 60-cm long and 12-cm wide arms were equally spaced and attached to a central platform. The experiment began 7 days after the Morris water maze test. Rats are restricted to food access during the experiment to maintain 80–85% of their baseline body weight. They were then placed in the maze for 20 min a day for habituation. Throughout the maze, 45 mg food pellets (Neuroscience Inc., Tokyo, Japan) were scattered for the rats to eat. The habituation sessions were carried out for 2 days. From the day after the habituation, they were trained in the working memory task two trials a day for 20 days. A 45 mg food pellet was placed in the food well at the edge of each arm. Transparent Plexiglas

guillotine doors, which could be operated by the experimenter by means of overhead lines, were placed between the center and each of the eight arms. The rat was placed in the center of the maze with all the guillotine doors closed. Then, all the doors were raised, and the rat was allowed to choose one of the arms. A choice was counted if the rat completely stepped into the arm, and then all the doors were closed. An error was counted if the rat reentered the arm that had been visited previously in the trial. This procedure was continued until all eight arms had been chosen or 5 min had passed since the start of the trial.

1.5.3. Open field test

A 90-cm × 90-cm open field apparatus (O'Hara, Tokyo, Japan) was used. The brightness at the center was 50 lux. Each rat was placed in a certain corner of the apparatus and then allowed to move freely during a 10-min session daily for 7 consecutive days. The behavior of the rats was recorded by a CCD camera. The distance traveled during a session and the stay time in the central area (36% of the field) were assessed to characterize spontaneous locomotor activity and anxiety-like behavior. Furthermore, we measured duration per movement by automated software TimeOFCR1 (O'Hara)

1.5.4. Novel object recognition test

On the day following the 7-day open field sessions, we carried out the novel object

recognition test[8] inside the same apparatus. First, two identical objects (cuboid) were placed diagonally in the open field. Then, the rats were allowed to explore the objects for 3 min. After the exploration, the apparatus and the objects were wiped with 70% ethanol, and one of the objects was replaced with a different and unfamiliar object (sphere). Which of the cuboids should be replaced was determined randomly for each rat. The rat was placed again in the apparatus after a 5-min interval, and the time of approach to each object was recorded for another 3 min.

1.5.5. Social interaction test

After more than one week from the novel object recognition test, the social behaviors of the rats were tested. We slightly modified our previous protocol (Fujiahra et al., 2015)[1] as described below. An open field apparatus was also used here. Two identical wire cages were placed inside the arena diagonally. In the habituation session, rats were allowed to move freely around the arena for 10 min. A stranger rat was enclosed in one of the wire cages immediately after the habituation. Then, the rat to be tested was put back on the arena (sociability test). The times the rats stayed around the cages were counted during a 10-min session. After a 5-min interval, we placed a different stranger rat in the empty wire cage, and again, the time spent around each cage was recorded for 10 min (social novelty preference test).

1.5.6. Y-maze test

The Y-maze apparatus for rats (O’Hara, Tokyo, Japan) was used to evaluate spatial working memory without food restriction stress. The length of each arm was 60 cm, and its width was 6 cm. The center of the maze was illuminated at 50 lux. Each rat was placed at the end of one arm and allowed to move freely through the maze for 8 min. The spontaneous alternation rate was calculated by dividing the total possible alternations by the number of actual alternations.

1.5.7. Elevated plus maze test

The apparatus consisted of two open arms and two closed arms (O’Hara, Tokyo, Japan). Each arm was of the same size (10 cm × 50 cm), and the closed arm had 35-cm-high transparent walls. The apparatus was placed 50 cm above the floor. The brightness on the central square of the maze was 50 lux. The rats were allowed to move freely in the maze for 10 min. The durations spent in the closed and open arms were measured.

1.5.8. Acoustic startle response and prepulse inhibition test

The experiment was carried out as described previously (Fujihara et al., 2015) [1]. The acoustic startle response was evaluated with nine different intensities (70, 75, 80, 85, 90, 95, 100, 110, and 120 dB). The white noise stimuli (40 ms) at each intensity were presented in quasi-

random order and at random intertrial intervals (10–20 s). In the prepulse inhibition (PPI) session, rats experienced five different types of stimuli: no stimulus; startle stimulus (120 dB, 40 ms) only; prepulse 70 dB (20 ms, lead time 100 ms) and pulse 120 dB; prepulse 75 dB (20 ms, lead time 100 ms) and pulse 120 dB; and prepulse 80 dB (20 ms, lead time 100 ms) and pulse 120 dB. Each trial was repeated 10 times in quasi-random order with random intertrial intervals (10–20 s). PPI was defined as the percent decline in the startle response: 100 − [(startle amplitude after prepulse and pulse)/(startle amplitude after pulse only)] × 100.

1.5.9. Porsolt forced Swim test

Plexiglas cylinders (20 cm diameter × 45 cm height; O'Hara, Tokyo, Japan) were filled with water, which was maintained at 22 ± 2° C. Each rat was placed in the water, and behavior was recorded for 10 min by a CCD camera. The immobility time was counted every 2 min during the session by a trained experimenter. We repeated the same experiment for 2 days.

1.5.10. Locomotor activity after MK 801 injection

The same apparatus used for the open field test was also used for this experiment. Each rat was placed in a certain corner of the open field, and its behavior was recorded using a video camera. At 30 min after the start of the test, MK-801 (0.2 mg/kg; Sigma-Aldrich, St. Louis, MO, USA), an NMDA-type glutamate receptor antagonist, was injected intraperitoneally.

Then, the rat was returned to the apparatus for another 150 min. The distance traveled during the session was quantified.

1.6 Data analysis

Graphs were generated using GraphPad Prism 5 (GraphPad Software, Inc., La Jolla, CA, USA). Collected data were analyzed using the statistical software R (https://www.r-project.org/) [9].

1.7 References for Supplementary Materials and Methods

1 Fujihara, K. et al. Glutamate decarboxylase 67 deficiency in a subset of GABAergic neurons induces schizophrenia-related phenotypes. *Neuropsychopharmacology* **40**, 2475–2486 (2015)

2 Hanamura, K. et al. Low accumulation of drebrin at glutamatergic postsynaptic sites on GABAergic neurons. *Neuroscience* **169**, 1489–1500 (2010)

3 Suto, T., Kato, D., Obata, H & Saito, S. Tropomyosin receptor kinase B receptor activation in the locus coeruleus restores impairment of endogenous analgesia at a late stage following nerve injury in rats. *J. Pain* **20**, 600–609 (2019)

4 Nunez, J. Morris water maze experiment. *J. Vis. Exp.* **19**, pii 897 (2008)

5 Morris, R.G., Garrud, P., Rawlins, J.N. & O'Keefe, J. Place navigation impaired in rats with hippocampal lesions. *Nature* **297**, 681–683 (1982)

6 Olton, D.S. & Samuelson, R.J. Remembrance of places passed: Spatial memory in rats. *J. Exp. Psychol. Anim. Behav. Process.* **2**, 97–116 (1976)

7 Kawabe, K., Iwasaki, T. & Ichitani, Y. Repeated treatment with N-methyl-D-aspartate antagonists in neonatal, but not adult, rats causes long-term deficits of radial-arm maze learning. *Brain Res.* **1169**, 77–86 (2007)

8 Antunes, M. & Biala, G. The novel object recognition memory: Neurobiology, test procedure, and its modifications. *Cogn. Process* **13**, 93–110 (2012)

9 R-CoreTeam. A language and environment for statistical computing. Vienna, Austria: R foundation for Statistical Computing; http://www.R-project.org. (2017)

2. Supplementary Figures

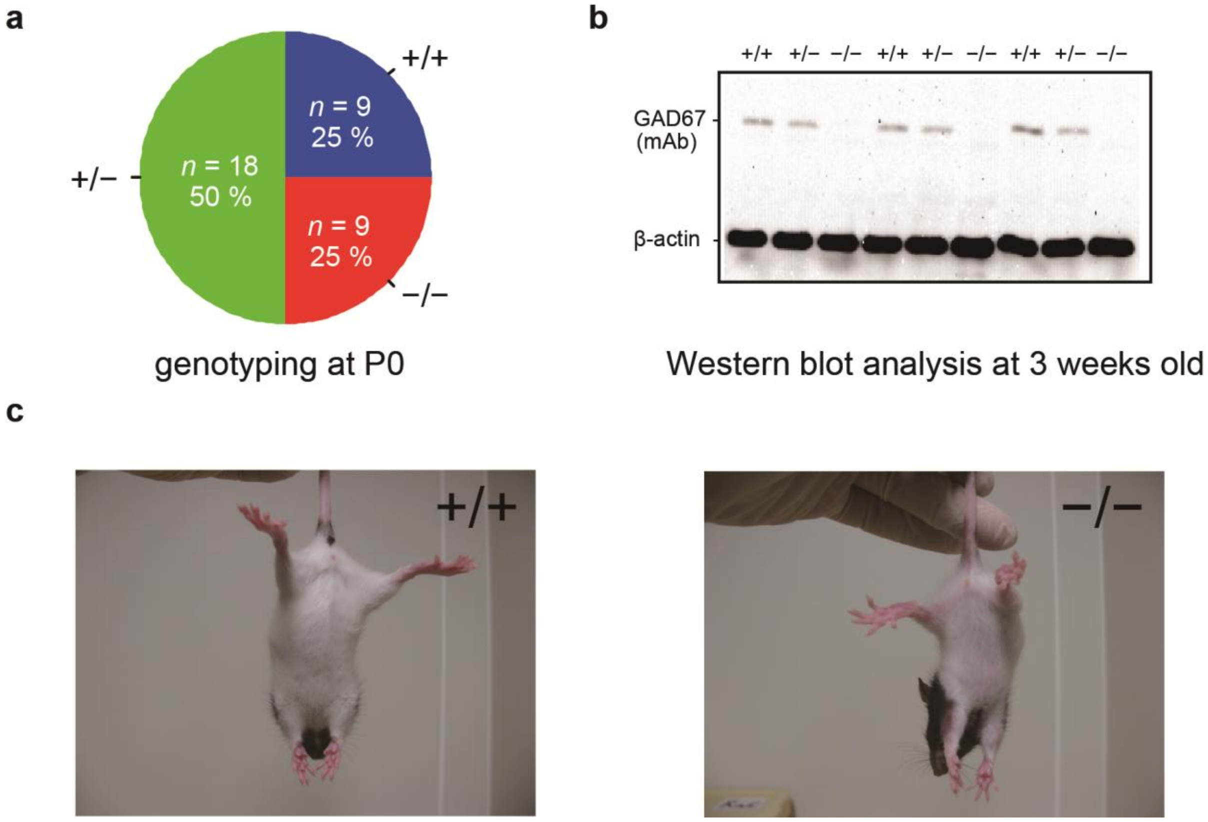

Supplementary Fig. 1 Further details on the generation of the *Gad1* knockout rat

a Frequency of each genotype on the first day after birth. In this example, the observed distribution was equal to the theoretical expectation based on Mendel's law. **b** Western blot analysis using a monoclonal antibody specific to GAD67 protein, showing that GAD67 was undetectable in the neocortex of *Gad1*$^{-/-}$ rats at 3 weeks of age (*n* = 3 in each genotype). **c** *Gad1*$^{-/-}$ rats showed no ataxia-like behavior. Neither *Gad1*$^{-/-}$ nor *Gad1*$^{+/+}$ rats displayed hindlimb clasping at 3 weeks of age.

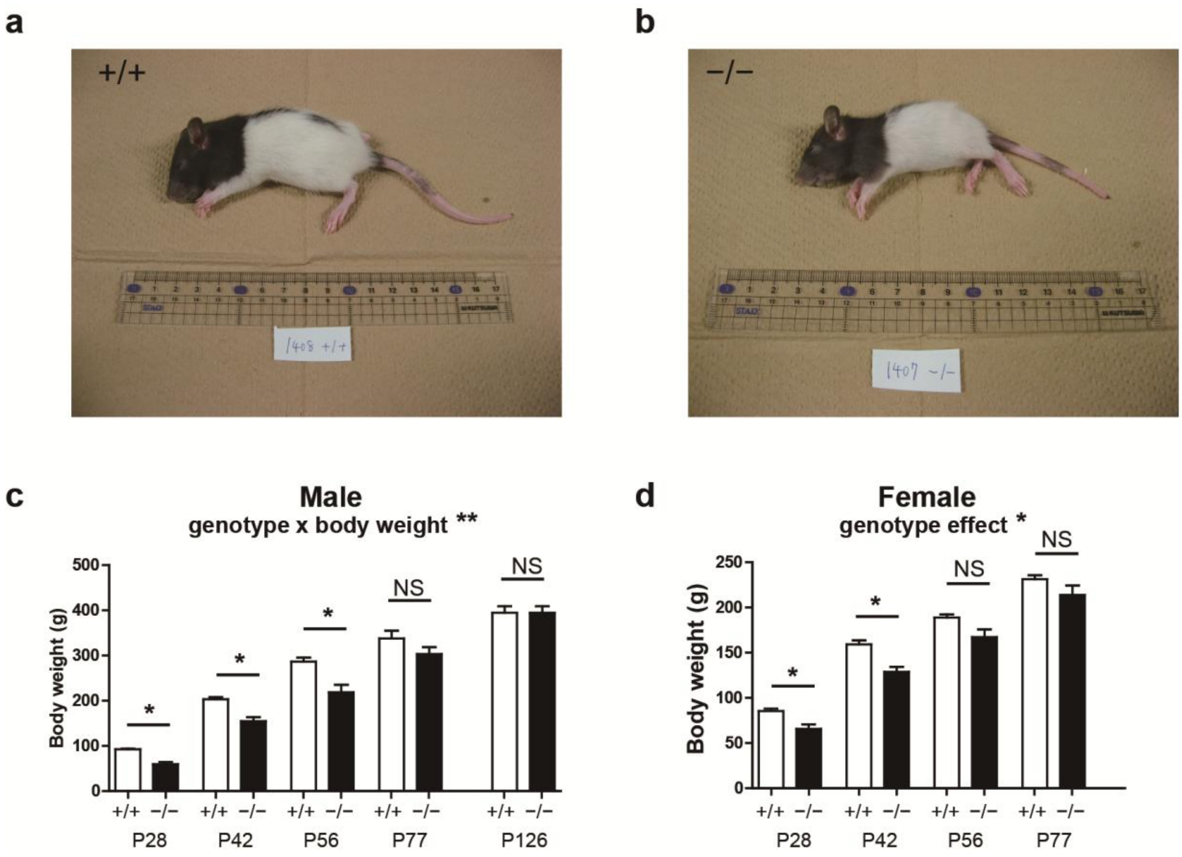

Supplementary Fig. 2   Growth retardation in *Gad1*$^{-/-}$ rats

a and **b** Representative pictures of *Gad1*$^{+/+}$ and *Gad1*$^{-/-}$ rats at P24, respectively. The body size of the *Gad1*$^{-/-}$ rats was smaller than that of the *Gad1*$^{+/+}$ rats. **c** Male *Gad1*$^{-/-}$ rats showed lower body weight compared with *Gad1*$^{+/+}$ littermates until P56 (effect of genotype, $F(1, 6) = 7.6476$, $p < 0.05$; age, $F(4, 24) = 470.1832$, $p < 0.001$; genotype × age, $F(4, 24) = 4.8277$, $p < 0.01$; *post hoc* test adjusted by Holm's method, P28, $p < 0.01$; P42, $p < 0.05$; P56, $p < 0.05$). As the bodyweight of *Gad1*$^{-/-}$ rats became comparable to WT from P77, their growth pattern showed catch-up growth. **d** Female *Gad1*$^{-/-}$ rats also showed a similar reduction in body weight as males (effect of genotype, $F(1, 10) = 9.0632$, $p < 0.05$). *$p < 0.05$; NS, not significant.

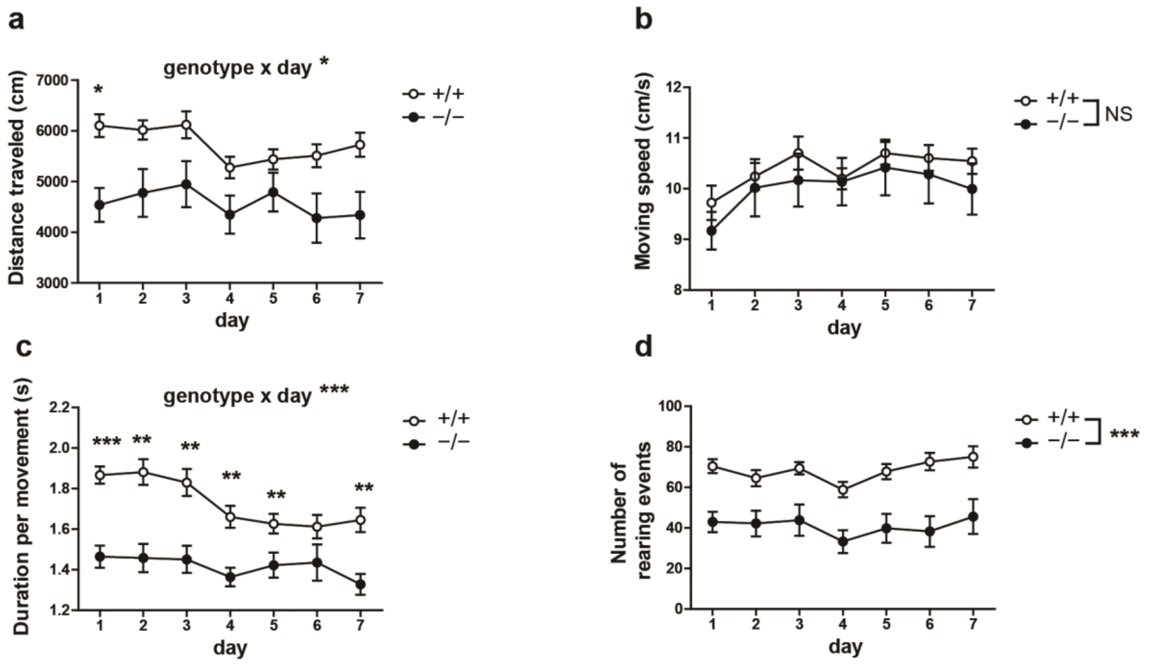

Supplementary Fig. 3 Time course of parameters measured in the open field test

a Distance traveled was significantly lower in *Gad1*$^{-/-}$ rats (effect of genotype, $F(1,22) = 6.9021$, $p < 0.05$; effect of day, $F(6, 132) = 7.2965$, $p < 0.001$; genotype × day interaction, $F(6, 132) = 2.2782$, $p < 0.05$; *post hoc* test adjusted by Holm's method, day 1, $p < 0.01$). **b** The moving speed of *Gad1*$^{-/-}$ rats was similar to that of *Gad1*$^{+/+}$ rats (effect of genotype, $F(1, 22) = 0.4586$, $p = 0.5053$). **c** The average duration of each movement of *Gad1*$^{-/-}$ rats was dramatically shorter than that of *Gad1*$^{+/+}$ rats (effect of genotype, $F(1,22) = 20.6206$, $p < 0.001$; effect of day, $F(6, 132) = 8.7301$, $p < 0.001$; genotype × day interaction, $F(6, 132) = 3.1567$, $p < 0.01$; *post hoc* test adjusted by Holm's method, day 1, $p < 0.001$; day 2, $p < 0.01$; day 3, $p < 0.01$; day 4, $p < 0.01$; day 5, $p < 0.01$; day 7, $p < 0.01$). **d** *Gad1*$^{-/-}$ rats reared less frequently than *Gad1*$^{+/+}$ rats (effect of genotype, $F(1, 22) = 14.8694$, $p < 0.001$). *$p < 0.05$, **$p < 0.01$, ***$p < 0.001$; NS, not significant.

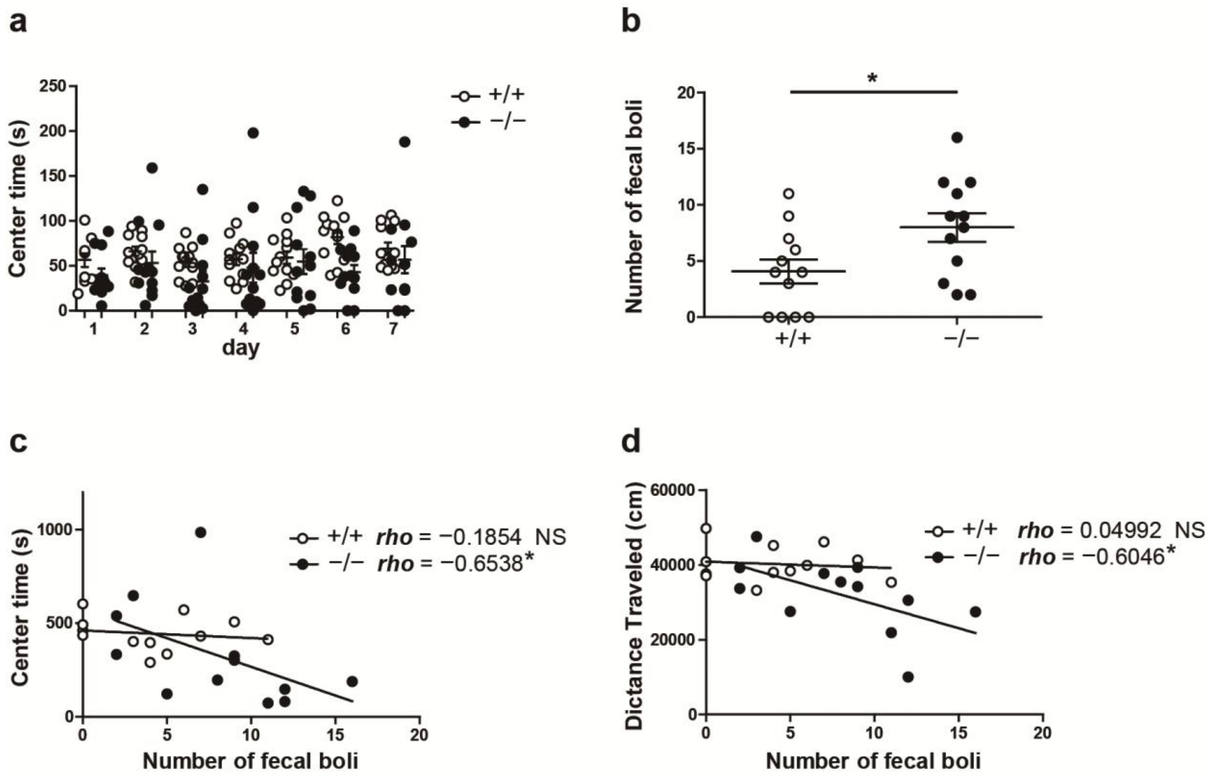

Supplementary Fig. 4 Decreased center time and number of fecal boli in the open field

a Time course of the center time during the open field test. The summed time of staying in the center region was significantly lower in *Gad1*$^{-/-}$ rats (see Fig. 3e). **b** The total number of fecal boli during the open field session was significantly higher in *Gad1*$^{-/-}$ rats than in *Gad1*$^{+/+}$ rats ($t(21.407) = 2.3492$, $p < 0.05$). **c** The number of fecal boli was negatively correlated with the center time only in *Gad1*$^{-/-}$ rats (Spearman's rank correlation; *Gad1*$^{+/+}$, $rho = -0.185$, $p = 0.564$; *Gad1*$^{-/-}$, $rho = -0.654$, $p < 0.05$). **d** The number of fecal boli was also negatively correlated with the distance traveled only in *Gad1*$^{-/-}$ rats (Spearman's rank correlation; *Gad1*$^{+/+}$, $rho = 0.0499$, $p = 0.8776$; *Gad1*$^{-/-}$, $rho = -0.605$, $p < 0.05$). *$p < 0.05$; NS, not significant.

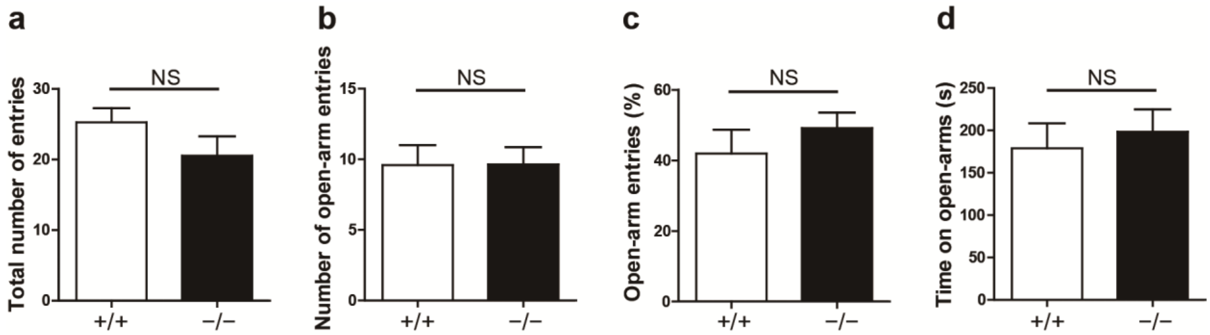

Supplementary Fig. 5 Anxiety-like behaviors in the elevated plus maze

The total number of entries to arms was similar in both genotypes ($t(20.741) = 0.0283$, $p = 0.9777$) **(a)**. Neither the number of entries into open arms ($t(20.913) = 0.4862$, $p = 0.6319$) **(b)**, the percentage of open-arm entries ($t(18.631) = 0.8814$, $p = 0.3893$) **(c)**, nor the time spent on the open arms ($t(18.698) = –1.3884$, $p = 0.1813$) **(d)** was altered in *Gad1*$^{–/–}$ rats compared to *Gad1*$^{+/+}$ rats. The distance traveled on the maze was significantly decreased in *Gad1*$^{–/–}$ rats, as in the open field test ($t(20.426) = –2.5658$, $p < 0.05$) (graph not shown). NS, not significant.

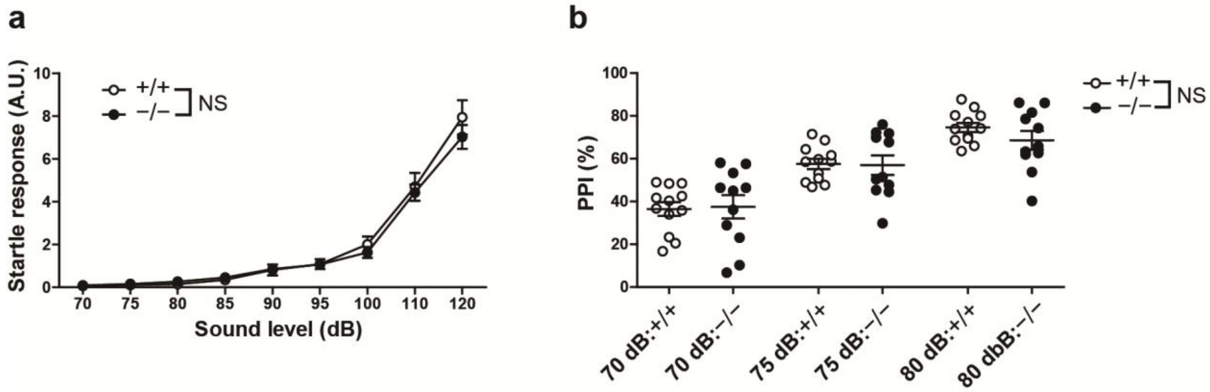

Supplementary Fig. 6 Normal acoustic startle response and sensorimotor gating

Gad1$^{-/-}$ rats showed a similar acoustic startle response to *Gad1*$^{+/+}$ rats (main effect of genotype, $F(1, 21) = 0.2544$, $p = 0.6192$) (*Gad1*$^{+/+}$, $n = 12$; *Gad1*$^{-/-}$, $n = 11$). There was a dose-dependent increase in the startle amplitude in both genotypes (effect of stimulus, $F(8, 168) = 155.9299$, $p < 0.001$). We then assessed deficits in sensorimotor gating using the prepulse inhibition (PPI) test. *Gad1*$^{-/-}$ rats did not display any alterations in PPI compared with *Gad1*$^{+/+}$ (main effect of genotype, $F(1, 21) = 0.1632$, $p = 0.6903$; prepulse, $F(2, 42) = 113.2125$, $p < 0.001$). NS, not significant.

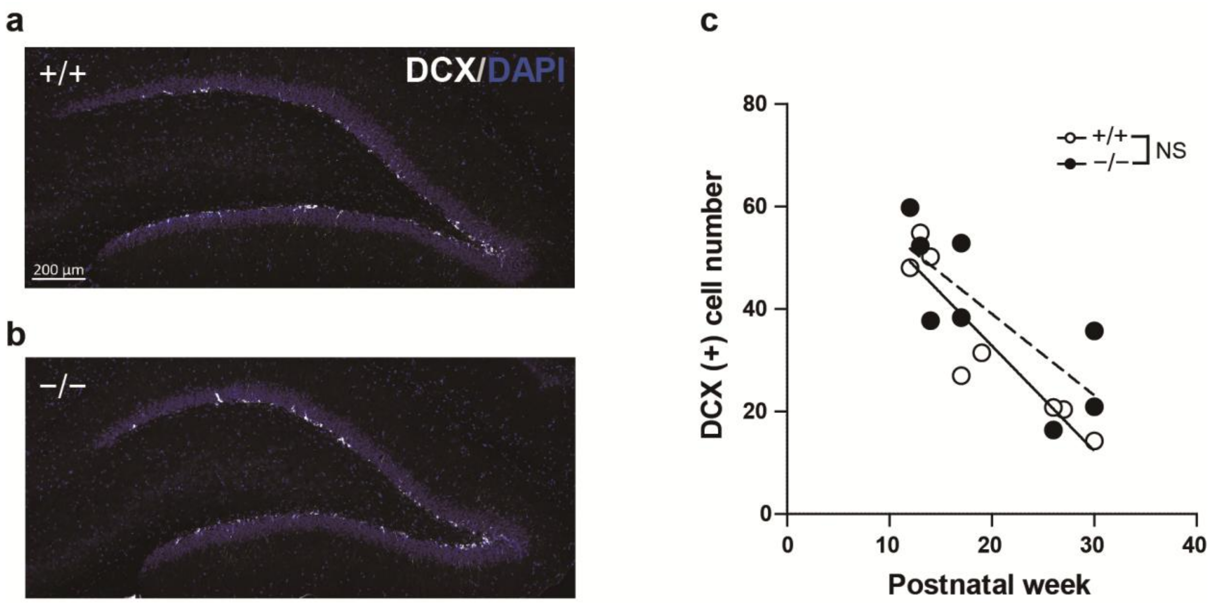

Supplementary Fig. 7 Normal adult neurogenesis in the dentate gyrus of *Gad1*$^{-/-}$ rats

a–**b** Representative doublecortin (DCX) staining of *Gad1*$^{+/+}$ and *Gad1*$^{-/-}$ rats, respectively. **c** Both genotypes displayed age-dependent reduction in the number of DCX-positive cells in the dentate gyrus (ANCOVA, age, $t(12) = –3.716$, $p < 0.01$; genotype × age, $t(12) = –0.735$, $p = 0.47658$). There was no difference in the number of DCX-positive cells between the two genotypes after controlling for the confounding effect of age ($t(13) = –1.451$, $p = 0.170$) (*Gad1*$^{+/+}$, $n = 8$; *Gad1*$^{-/-}$, $n = 8$). NS, not significant.
